# The Impact of Major Depressive Disorder on Somatic and Psychiatric Outcomes Following Elective Single-Level Lumbar Fusion: A Propensity Score-Matched Analysis

**DOI:** 10.1177/21925682261445029

**Published:** 2026-04-24

**Authors:** Sri Tummala, Hetsinhji Chavda, Tarun R. Sontam, David C. Gibbs, Ioannis Avramis, James M. Rizkalla

**Affiliations:** 1 Department of Orthoapaedic Surgery, Texas A&M College of Medicine, College Station, TX, USA; 2 Department of Orthoapedic Surgery, 22683 Baylor University Medical Center, Dallas, TX, USA; 3 Baylor Scott & White Spine & Scoliosis Center, 22683 Baylor University Medical Center, Dallas, TX, USA

**Keywords:** major depressive disorder, lumbar fusion, spine surgery outcomes, psychiatric comorbidity, mental health, optimization, depression

## Abstract

**Study Design:**

Retrospective database study.

**Objective:**

Major depressive disorder (MDD) is common psychiatric comorbidity among lumbar fusion candidates, yet its association with postoperative psychiatric and somatic symptoms morbidity and healthcare utilization remains inadequately characterized. This study evaluated whether preoperative MDD increases the risk of adverse psychiatric and medical outcomes following single-level lumbar fusion.

**Methods:**

Utilizing a national electronic health record network, adults undergoing elective single-level lumbar fusion between 2015 and 2025 were identified. Patients with recurrent MDD diagnosed within 1-year preoperatively were compared with patients without MDD after one-to-one propensity score matching for demographics, comorbidities, laboratory values, and preoperative medications. Outcomes were assessed at 90-days and 1-year.

**Results:**

The matched cohort included 11 570 patient pairs. Patients with MDD demonstrated higher rates of newly diagnosed psychiatric conditions at both time points, including generalized anxiety disorder, adjustment disorder, post-traumatic stress disorder, alcohol-use disorder, and dementia; opioid-use disorder was increased at 1 year. MDD was also associated with higher rates of postoperative somatic symptom diagnoses, including chest pain, dizziness, and shortness of breath. Emergency department utilization did not differ at 90 days but was higher at 1 year, while inpatient readmission rates were increased at both time points. There were no differences in reoperation rates or mortality between cohorts.

**Conclusions:**

Preoperative MDD was associated with postoperative psychiatric morbidity, somatic symptoms, and increased healthcare utilization within 1 year following the procedure, without evidence of higher reoperation or mortality. These findings support routine depression screening and perioperative mental health optimization in single-level lumbar fusion candidates.

## Introduction

Major depressive disorder (MDD) is a prevalent psychiatric condition defined by the Diagnostic and Statistical Manual of Mental Disorders, Fifth Edition (DSM-5) as the presence of persistent depressed mood or anhedonia accompanied by additional cognitive, behavioral, and somatic symptoms for at least 2 weeks.^1^ In clinical practice, MDD is most often identified through validated screening instruments such as the Patient Health Questionnaire (PHQ-9) or structured clinical interviews and is documented in electronic health records using standardized diagnostic codes.^1,2^ MDD affects nearly 10% of adults in the United States and is projected by the World Health Organization to become the leading global contributor to disease burden by 2030.^2,3^ Despite growing recognition of its clinical impact, depression remains frequently underdiagnosed and undertreated in surgical populations, where symptoms may be obscured by chronic pain, physical disability, or perioperative stress.^2,3^ Accordingly, the U.S. Preventive Services Task Force recommends routine depression screening for all adults, citing established associations with impaired recovery, increased medical morbidity, and elevated mortality when left unmanaged.^2^

Patients with musculoskeletal and degenerative spine conditions exhibit a disproportionately high prevalence of depressive disorders.^3-5^ Among individuals with chronic low back pain and lumbar degenerative disease, reported depression prevalence rates range from approximately 20% to 31%, substantially exceeding those observed in the general population.^3-5^ These conditions are closely associated with psychological distress, functional impairment, and diminished quality of life, which may contribute to or exacerbate underlying depressive symptoms.^4,5^ Importantly, MDD frequently co-occurs with other psychiatric conditions such as anxiety disorders, substance use disorders, and post-traumatic stress disorder, creating considerable diagnostic overlap that complicates efforts to isolate the independent contribution of any single condition to postoperative outcomes.^4,5^ Accordingly, the present study focused exclusively on patients with MDD while excluding those with other concurrent psychiatric diagnoses, thereby reducing confounding from overlapping conditions and enabling a more precise estimation of the independent perioperative risk attributable to MDD.

Lumbar fusion is a commonly performed procedure aimed at alleviating pain, restoring stability, and improving function in patients with degenerative spinal disease. Although many patients experience substantial postoperative improvement, a meaningful subset report persistent symptoms, dissatisfaction, or ongoing health care utilization following surgery.^6^ Increasing evidence indicates that depressive disorders independently influence postoperative outcomes across orthopaedic procedures, extending beyond procedure-specific complications to include cardiopulmonary events, infection, cerebrovascular disease, venous thromboembolism, and increased emergency and inpatient utilization.^7-9^ In the spine surgery literature, a recent systematic review and meta-analysis found that preoperative depression was associated with worse postoperative outcomes following lumbar fusion, including lower rates of functional improvement and higher rates of persistent disability.^8^ The unique psychological stressors associated with chronic back pain, including fear of neurological compromise, prolonged disability, and uncertainty surrounding functional recovery, may further heighten the vulnerability of patients with depression undergoing spine procedures.^4-6^ Depression has also been associated with prolonged hospital length of stay, higher rates of early readmission, and increased perioperative health care costs.^8,10^ Additionally, postoperative stress may precipitate new-onset psychiatric conditions, including anxiety and substance use disorders, underscoring the bidirectional relationship between psychological health and postoperative recovery.^9^

Despite these observations, contemporary perioperative risk stratification models often inadequately account for psychiatric comorbidities, potentially underestimating their impact on postoperative morbidity and health care utilization.^10^ Although prior studies suggest that patients with depression can achieve meaningful postoperative improvements in pain and function following spine surgery, these outcomes are frequently influenced by baseline mental health status and psychosocial factors.^10,11^ Accordingly, the presence of depression alone should not necessarily preclude surgical intervention, as several investigations have reported comparable postoperative functional improvements among carefully selected patients with preoperative depression compared with those without depression.^12,13^ Nevertheless, patients with coexisting depression and poor baseline mental health remain at increased risk for persistent symptoms, dissatisfaction, and postoperative complications, even after technically successful procedures, underscoring the importance of comprehensive preoperative assessment and management.^11^

Although the impact of MDD on postoperative outcomes has been extensively studied in joint arthroplasty populations,^7,8,10,11^ its independent influence in the setting of spine surgery remains insufficiently defined. Prior spine studies have focused primarily on patient-reported outcome measures, have frequently grouped MDD with other psychiatric conditions rather than isolating its independent effect, have not comprehensively evaluated postoperative healthcare utilization patterns, and have generally lacked propensity score-matched designs to account for baseline differences between affected and unaffected populations.^10,12,16^ Given the rising prevalence of depression and increasing utilization of lumbar fusion, further investigation is warranted to clarify the role of isolated MDD in postoperative outcomes following the procedure. Accordingly, the purpose of this study was to evaluate the association between preoperative MDD and psychiatric and somatic postoperative outcomes following single-level lumbar fusion to help inform preoperative counseling, risk stratification, and multidisciplinary perioperative care for clinicians.

## Methods

### Data Source

This retrospective cohort study utilized the TriNetX US Collaborative Network (TriNetX, Cambridge, Massachusetts), a federated database aggregating de-identified electronic health record (EHR) data from participating healthcare organizations across the United States. The platform provides longitudinal data on patient demographics, diagnoses, procedures, medications, and laboratory values, with full compliance with the Health Insurance Portability and Accountability Act (HIPAA). As the dataset contains no identifiable patient information, institutional review board approval was not required.

### Inclusion and Exclusion Criteria

**Figure 1.**
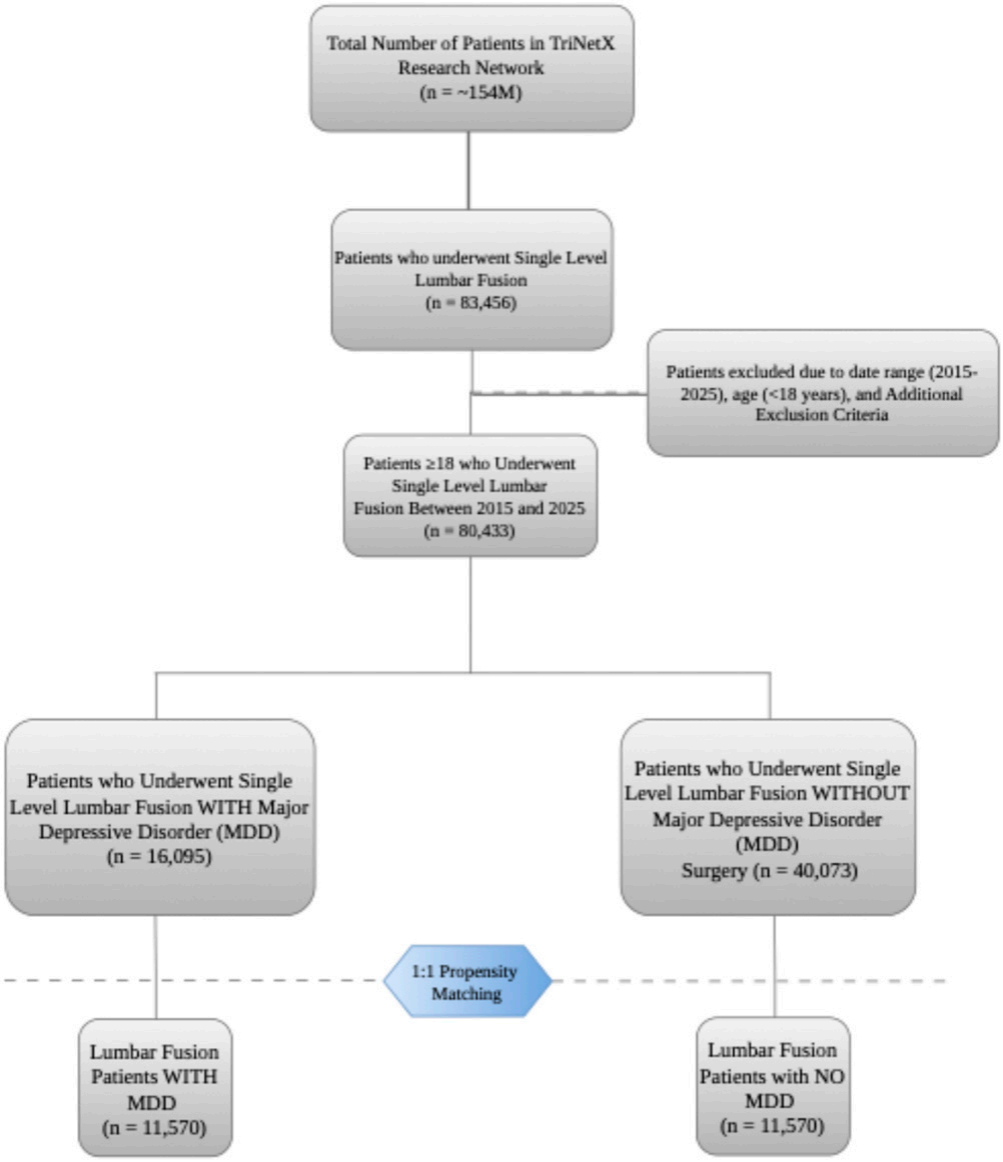
STROBE flowchart for patient selection

The study population included adults (≥18 years) who underwent elective single-level lumbar fusion between January 1, 2015, and January 1, 2025. The exposure group consisted of patients with a documented International Classification of Diseases, 10th Revision (ICD-10) diagnosis of major depressive disorder, recurrent type (F33) within 1 year prior to surgery. The study was restricted to recurrent MDD (F33) rather than including single-episode MDD (F32) in order to capture patients with an established and persistent depressive illness more likely to reflect chronic psychiatric burden during the perioperative period. Exposure was defined as the presence of at least one coded encounter with an F33 diagnosis, with inpatient and outpatient diagnoses treated equally. The control group comprised patients undergoing the same procedure without any recorded history of MDD. Patients with a preoperative diagnosis of neoplasm, trauma, infection, malunion, paralysis, spinal cord injury, or other psychiatric or neurocognitive conditions including bipolar disorder, schizophrenia, anxiety disorders, substance use disorders, and dementia were excluded from both cohorts. This study was conducted in accordance with the Strengthening the Reporting of Observational Studies in Epidemiology (STROBE) guidelines. A detailed illustration of patient selection and cohort construction is presented in Figure 1. The study cohort included patients undergoing single-level lumbar fusion as identified by Current Procedural Terminology (CPT) codes corresponding to single-interspace procedures (Appendix A). Both instrumented and non-instrumented fusion techniques were included, and anterior, posterior, and lateral approaches were analyzed together. The TriNetX platform does not reliably distinguish between minimally invasive and open surgical techniques based on available procedural codes; this was acknowledged as a limitation. Revision procedures were excluded from the index cohort to limit the analysis to primary surgical episodes. Procedural heterogeneity is acknowledged as a potential source of variability in outcomes.

### Propensity Score Matching

To reduce baseline imbalances between cohorts, 1:1 propensity score matching (PSM) was performed using clinically relevant variables. Demographic factors, including age, sex, race, and ethnicity, were included given their established associations with postoperative complications, readmissions, and recovery following major orthopaedic procedures.^9,11^ Medical comorbidities were selected based on their known or suspected influence on perioperative risk and outcomes following spine surgery and included hypertension, hyperlipidemia, diabetes mellitus, chronic ischemic heart disease, chronic kidney disease, tobacco and nicotine use, hypothyroidism, polycystic ovarian syndrome, prior cerebral infarction, and obesity.^9,11^ Medication exposure within the preoperative period, including opioids, antidepressants, antipsychotics, and gabapentinoids, was incorporated into the matching algorithm due to their frequent use among patients with chronic pain or psychiatric conditions and their documented associations with adverse postoperative outcomes such as respiratory complications, prolonged recovery, and increased health care utilization.^11,15^ In addition, body mass index (BMI) and hemoglobin A1c (HbA1c) values were included given their clinical relevance, as elevated BMI has been associated with increased perioperative risk in spine surgery, while HbA1c serves as a marker of glycemic control and predictor of surgical site complications following orthopaedic procedures.^15^ All diagnostic, procedural, and medication codes used for matching are detailed in Appendix A. Propensity scores were estimated using logistic regression, and 1:1 nearest-neighbor greedy matching was performed using a caliper width of 0.1 standard deviations of the logit of the propensity score, without replacement. Patients without available BMI or HbA1c values were not excluded from the overall cohort; however, matching on these variables was performed only among patients for whom values were recorded. No trimming or common-support restrictions were applied beyond the caliper constraint. Preoperative medication exposure was defined as at least 1 prescription documented on the day of the index procedure. Dose and duration data were not available within the TriNetX platform.

Prior to PSM, patients with MDD exhibited greater prevalence of cardiometabolic disease, chronic kidney disease, and tobacco use, as well as substantially higher utilization of opioids, antidepressants, and gabapentinoids in the preoperative period (all *P* < 0.001).

After PSM, standardized mean differences (SMD) for all matched covariates fell below 0.1, indicating negligible residual imbalance (Table 2). No clinically meaningful differences remained in age, sex, race, ethnicity, or major medical comorbidities. Although BMI and HbA1c values differed slightly between groups, these differences were statistically but not clinically significant. Complete baseline characteristics before and after matching are presented in Tables 1 and 2.

**Table 1.** Patient Demographics and Characteristics Before Propensity-Score Matching

Characteristic	Patient count n (% of cohort)	Patient count n (% of cohort)	*P*-value	SMD
Before matching	16,095	40,073		
Age at index in years^a^	59.0 ± 13.0	61.1 ± 13.8	<0.001	0.158
White	13 638 (84.7%)	32 898 (82.1%)	<0.001	0.071
Black or African American	1474 (9.2%)	3366 (8.4%)	0.004	0.027
Other race	393 (2.4%)	1069 (2.7%)	0.128	0.014
Hispanic or Latino	814 (5.1%)	2002 (5.0%)	0.762	0.003
Male	4977 (30.9%)	20 591 (51.4%)	<0.001	0.425
Female	11 116 (69.1%)	19 474 (48.6%)	<0.001	0.425
Chronic ischemic heart disease	2739 (17.0%)	4873 (12.2%)	<0.001	0.138
Diabetes mellitus	4392 (27.3%)	7088 (17.7%)	<0.001	0.231
Primary hypertension	9988 (62.1%)	18 705 (46.7%)	<0.001	0.313
Tobacco use	1419 (8.8%)	1238 (3.1%)	<0.001	0.244
Nicotine use	3994 (24.8%)	4475 (11.2%)	<0.001	0.361
Hyperlipidemia	7278 (45.2%)	11 994 (29.9%)	<0.001	0.320
Overweight and obesity	6393 (39.7%)	8277 (20.7%)	<0.001	0.425
Hypothyroidism	3312 (20.6%)	4233 (10.6%)	<0.001	0.279
Polycystic ovarian syndrome	185 (1.1%)	101 (0.3%)	<0.001	0.108
Chronic Kidney Disease (CKD)	1772 (11.0%)	2641 (6.6%)	<0.001	0.156
Cerebral infarction	703 (4.4%)	839 (2.1%)	<0.001	0.129
Diseases of liver	2125 (13.2%)	2338 (5.8%)	<0.001	0.253
Antipsychotics use	5555 (34.5%)	4999 (12.5%)	<0.001	0.538
Antidepressant use	12 491 (77.6%)	9266 (23.1%)	<0.001	1.300
Opioid use	12 690 (78.8%)	23 457 (58.5%)	<0.001	0.449
Gabapentinoids use	10 444 (64.9%)	17 168 (42.8%)	<0.001	0.454
Skeletal muscle relaxants use	9794 (60.9%)	15 549 (38.8%)	<0.001	0.452
Nonsteroidal anti-inflammatory drugs (NSAIDs) use	5784 (35.9%)	9011 (22.5%)	<0.001	0.299
Hemoglobin A1c^a^	6.0 ± 1.1	6.1 ± 1.1	0.045	0.028
Body Mass Index (BMI)^a^	31.4 ± 6.7	30.1 ± 6.1	<0.001	0.188

SMD, standardized mean difference; BMI, body mass index.

^a^Value represents the mean age/BMI/hemoglobin A1c levels ± standard deviation.

**Table 2.** Patient Demographics and Characteristics After Propensity-Score Matching

Characteristic	Patient count n (% of cohort)	Patient count n (% of cohort)	*P*-value	SMD
After matching	11,570	11,570		
Age at index in years^a^	59.8 ± 13.1	59.7 ± 13.6	0.448	0.010
White	9784 (84.6%)	9827 (84.9%)	0.432	0.010
Black or African American	1014 (8.8%)	985 (8.5%)	0.497	0.009
Other race	284 (2.5%)	271 (2.3%)	0.576	0.007
Hispanic or Latino	540 (4.7%)	514 (4.4%)	0.412	0.011
Male	4323 (37.4%)	4145 (35.8%)	0.015	0.032
Female	7247 (62.6%)	7422 (64.1%)	0.017	0.031
Chronic ischemic heart disease	1739 (15.0%)	1678 (14.5%)	0.258	0.015
Diabetes mellitus	2646 (22.9%)	2569 (22.2%)	0.226	0.016
Primary hypertension	6567 (56.8%)	6399 (55.3%)	0.026	0.029
Tobacco use	612 (5.3%)	600 (5.2%)	0.723	0.005
Nicotine use	2066 (17.9%)	2085 (18.0%)	0.745	0.004
Hyperlipidemia	4550 (39.3%)	4383 (37.9%)	0.024	0.030
Overweight and obesity	3573 (30.9%)	3425 (29.6%)	0.034	0.028
Hypothyroidism	1862 (16.1%)	1832 (15.8%)	0.590	0.007
Polycystic ovarian syndrome	55 (0.5%)	63 (0.5%)	0.460	0.010
Chronic Kidney Disease (CKD)	1076 (9.3%)	1042 (9.0%)	0.438	0.010
Cerebral infarction	365 (3.2%)	359 (3.1%)	0.821	0.003
Diseases of liver	1088 (9.4%)	1044 (9.0%)	0.317	0.013
Antipsychotics use	2661 (23.0%)	2625 (22.7%)	0.573	0.007
Antidepressant use	7966 (68.9%)	7942 (68.6%)	0.734	0.004
Opioid use	8494 (73.4%)	8275 (71.5%)	0.001	0.042
Gabapentinoids use	6820 (58.9%)	6639 (57.4%)	0.016	0.032
Skeletal muscle relaxants use	6275 (54.2%)	6040 (52.2%)	0.002	0.041
Nonsteroidal anti-inflammatory drugs (NSAIDs) use	3611 (31.2%)	3415 (29.5%)	0.005	0.037
Hemoglobin A1c^a^	6.0 ± 1.1	6.0 ± 1.1	0.403	0.016
Body Mass index (BMI)^a^	30.8 ± 6.5	30.7 ± 6.4	0.390	0.013

SMD, standardized mean difference; BMI, body mass index.

^a^Value represents the mean age/BMI/hemoglobin A1c levels ± standard deviation.

### Cohort Selection

After application of the predefined inclusion and exclusion criteria, a total of 16 095 patients with a diagnosis of major depressive disorder and 40 073 control patients without MDD who underwent elective single-level lumbar fusion during the study period were identified. 1:1 PSM resulted in 11 570 well-matched patient pairs, which constituted the final analytic cohort for all subsequent comparisons.

### Outcomes Evaluated

Patients were followed for the development of prespecified psychiatric and medical outcomes evaluated at 90 days and 1 year following surgery. The 90-day interval was selected as the primary postoperative time point, as a substantial proportion of surgery-related adverse events occur within the first 31 to 90 days after orthopaedic procedures.^13^ One-year follow-up was included to assess the persistence of associations beyond the early postoperative period, as prior studies have demonstrated no significant differences in patient-reported outcomes between one- and 2-year follow-up after orthopaedic surgery.^14^ Psychiatric outcomes included new-onset diagnoses of generalized anxiety disorder, adjustment disorder, post-traumatic stress disorder, alcohol use disorder, opioid use disorder, and dementia. Somatic outcomes included all-cause mortality, emergency department utilization, inpatient readmission, reoperation, chest pain, dizziness, and shortness of breath. All outcomes were identified using ICD-10 and Current Procedural Terminology codes (Appendix A).

### Data Analyses

All figure creation and statistical analyses were performed using R (version 4.5.1; R Foundation for Statistical Computing, Vienna, Austria) and the built-in analytics tools available within the TriNetX platform. Risk ratios (RR) and 95% confidence intervals (CI) were generated by TriNetX, which internally computes risk ratios for binary outcomes rather than odds ratios. Statistical significance was defined as a two-tailed *P* < 0.05. Data extraction was performed on October 3, 2025. Only matched patient pairs were included in all post-matching analyses; unmatched patients were excluded. All-cause mortality was determined using the TriNetX “deceased” outcome flag derived from electronic health record documentation.

## Results

### Psychiatric Outcomes

**Figure 2.**
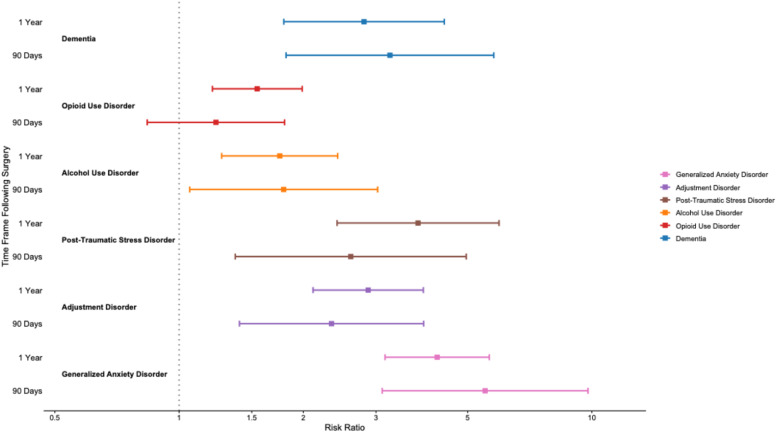
90-day and 1-year psychiatric outcomes following Lumbar Fusion

Patients with MDD exhibited significantly increased risk for multiple psychiatric conditions following single-level lumbar fusion at both 90 days and 1 year, as shown in Figure 2. At 90 days, higher risk was observed for generalized anxiety disorder (RR 5.51, 95% CI 3.11 to 9.79), adjustment disorder (RR 2.34, 95% CI 1.40 to 3.91), PTSD (RR 2.61, 95% CI 1.37 to 4.96), alcohol use disorder (RR 1.79, 95% CI 1.06 to 3.03), and dementia (RR 3.24, 95% CI 1.82 to 5.79). However, the risk for opioid use disorder was not significant (RR 1.23, 95% CI 0.84 to 1.80; *P* > 0.05).

At one year, all psychiatric associations remained significant for generalized anxiety disorder (RR 4.22, 95% CI 3.15 to 5.64), adjustment disorder (RR 2.87, 95% CI 2.11 to 3.91), PTSD (RR 3.79, 95% CI 2.41 to 5.96), alcohol use disorder (RR 1.75, 95% CI 1.27 to 2.42), opioid use disorder (RR 1.55, 95% CI 1.20 to 1.99), and dementia (RR 2.81, 95% CI 1.79 to 4.39) (*P* < 0.001 for all).

### Somatic Outcomes

**Figure 3.**
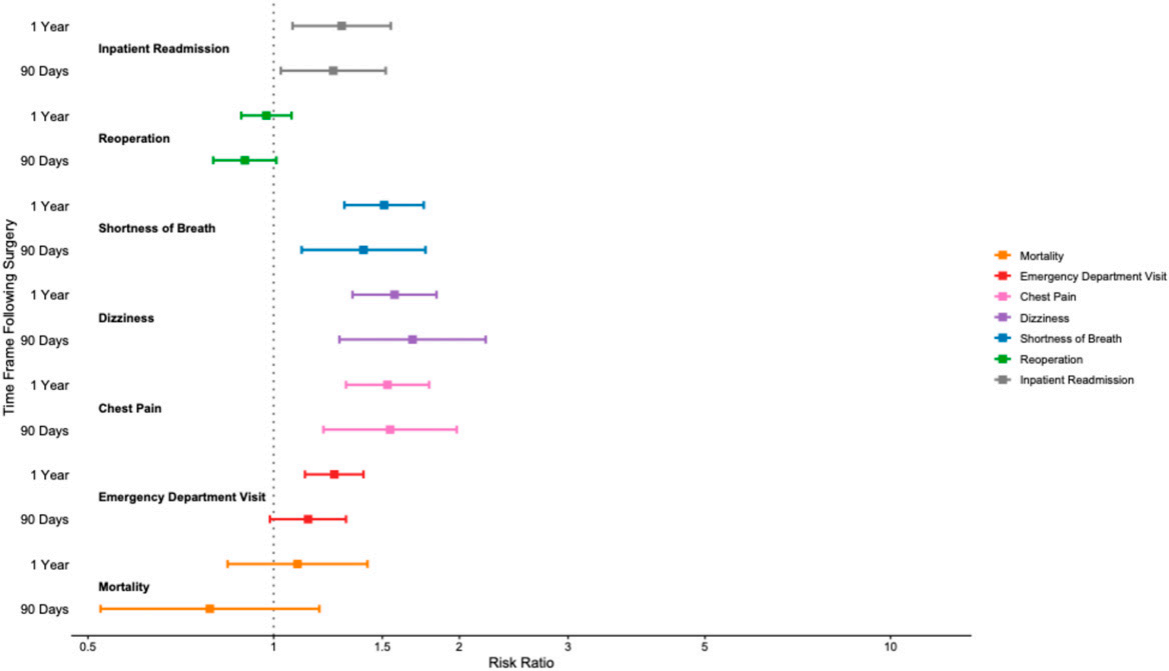
90-day and 1-year Somatic outcomes following Lumbar Fusion

As shown in Figure 3, patients with depression exhibited significantly increased risk of multiple somatic complications following single-level lumbar fusion at both 90 days and 1 year. At 90 days, MDD was associated with elevated risk of chest pain (RR 1.54, 95% CI 1.20 to 1.98), dizziness (RR 1.68, 95% CI 1.28 to 2.21), and shortness of breath (RR 1.40, 95% CI 1.11 to 1.76), while mortality (RR 0.79, 95% CI 0.52 to 1.19) and emergency department utilization (RR 1.14, 95% CI 0.99 to 1.31) were not significantly different.

At one-year, somatic risks remained significantly elevated for emergency department visit (RR 1.25, 95% CI 1.12 to 1.40), chest pain (RR 1.53, 95% CI 1.31 to 1.79), dizziness (RR 1.57, 95% CI 1.34 to 1.84), and shortness of breath (RR 1.51, 95% CI 1.30 to 1.75). However, mortality (RR 1.09, 95% CI 0.84 to 1.42) was not significantly associated with depression. All comparisons were statistically significant with *P* < 0.001 unless otherwise noted. Notably, the somatic outcomes assessed, chest pain, dizziness, and shortness of breath, represent symptom-based diagnoses identified through ICD-10 coding rather than confirmed structural cardiopulmonary complications. The absence of increased mortality or reoperation in the MDD cohort suggests a pattern of increased symptom burden and healthcare utilization rather than increased surgical or mechanical failure.

### Reoperations and Readmissions

In spine-specific exploratory analyses within the matched cohort, MDD was not associated with a higher risk of reoperation at 1 year (RR 0.97; 95% CI 0.89 to 1.07; *P* = 0.572) or at 90 days (RR 0.90, 95% CI 0.80 to 1.01). In contrast, MDD was associated with increased inpatient readmission at 90 days (RR 1.25; 95% CI 1.03 to 1.52) and 1 year (RR 1.29; 95% CI 1.07 to 1.55; *P* = 0.007).

## Discussion

Although single-level lumbar fusion is a highly effective procedure for alleviating pain and restoring function, its benefits may be less consistent among patients with psychiatric comorbidities, particularly depression.^15,16^ In this study, patients with major depressive disorder (MDD) demonstrated a higher associated risk of newly documented psychiatric diagnoses, somatic symptom diagnoses, substance use disorders, and greater postoperative healthcare utilization at both short- and long-term follow-up. Spine-specific procedural outcomes did not suggest greater mechanical failure, as the risk of reoperation at both evaluated time-intervals was similar to controls. These findings emphasize the importance of integrating mental health assessment and management into perioperative care pathways for spine surgery patients.

The greater psychiatric morbidity observed in this study underscores the perioperative period as a potentially destabilizing window for individuals with pre-existing mood disorders. Our findings align with broader orthopaedic literature, including studies in total joint arthroplasty, which report that depression is a robust predictor of medical complications, readmissions, and increased emergency department use.^7,8,17^ The substantial risks observed for generalized anxiety disorder, adjustment disorders, and PTSD suggest that the stress of a major spinal surgery may exacerbate underlying psychiatric vulnerabilities. Visser et al.^18^ reinforced this concept in a musculoskeletal context, reporting significantly poorer postoperative patient-reported outcomes among individuals with persistent depressive symptoms. The pathophysiological overlap between chronic pain and affective disorders likely creates a bidirectional vulnerability, where surgical stress can trigger or worsen psychiatric conditions in this patient population.^19^

Furthermore, patients with depression reported significantly higher somatic complications postoperatively, including chest pain, shortness of breath, and dizziness. Pan et al.^20^ highlighted that surgical patients with depression experience higher incidences of acute and chronic pain-related symptoms. Hecht et al.,^10^ in a systematic review, corroborated these findings, identifying increased pain perception and healthcare utilization in surgical patients with depression and anxiety. This elevated somatic burden and heightened pain sensitivity may explain the increased rates of ED visits and inpatient readmissions seen in our cohort, as patients may present more frequently with amplified or difficult-to-manage postoperative symptoms.

Patients with MDD also demonstrated a significantly increased risk of newly documented dementia diagnoses postoperatively. While meta-analyses have shown that a history of depression confers a higher long-term risk of developing dementia,^4,5,16^ the relatively short follow-up periods in the present study (90 days and 1 year) raise the possibility that these newly coded diagnoses may reflect recognition and documentation of previously undiagnosed cognitive impairment during increased postoperative clinical contact, rather than true new-onset neurodegeneration. Although speculative, activation of stress pathways and subsequent neuroinflammation during major surgery has been proposed as a potential mechanism linking depression to cognitive decline, which could be exacerbated in patients with underlying neural pathology associated with depression. However, this study design cannot evaluate causality, and these mechanistic hypotheses require prospective investigation.

Moreover, patients with pre-existing MDD were significantly more likely to have associated new-onset opioid and alcohol use disorders postoperatively. Diei et al.^21^ reported a substantially increased risk of persistent opioid use after orthopaedic surgery in patients with depression. Similarly, Etcheson et al.^22^ found higher postoperative opioid utilization in surgical patients with depression despite standardized analgesia, indicating altered pain perception or self-medication behaviors. The development of alcohol use disorder may follow a similar pattern, potentially as a maladaptive coping mechanism. Emerging evidence points to overlapping dysfunction in the brain’s reward and stress response systems as a mechanistic link between depression and substance use disorders.^23^

Contrary to findings in other surgical cohorts, mortality was not significantly elevated in this present study at either timepoint. This may be explained by the generally lower baseline mortality risk associated with elective spine surgery compared to other major procedures.^24,25^ However, the significant increases in ED visits, readmissions, and somatic complaints highlight a different pattern of risk, one centered on heightened morbidity and healthcare use rather than mortality. This pattern underscores a substantial burden on both the patient and the healthcare system.

These findings, together with prior work, are consistent with existing guideline recommendations supporting routine preoperative depression screening and timely intervention for patients undergoing lumbar fusion.^2,28^ Gordon et al.^26^ demonstrated that preoperative depression screening was associated with reduced postoperative complications and ED visits. A reasonable multidisciplinary approach could be to incorporate brief, standardized screening for all elective spine surgery candidates, using tools such as the PHQ-2 or PHQ-9, with positive screens prompting medication review, patient education, and, when appropriate, referral for optimization.^27,28^ For patients with active depression, a collaborative care model involving the surgeon, primary care physician, and mental health specialist could help mitigate perioperative risks.

### Limitations

This study has several limitations that warrant consideration. This study relies on EHR-based diagnostic coding, which may introduce misclassification; the specific diagnostic methods used by individual clinicians to establish MDD diagnoses could not be determined from the database. Restricting the exposure to recurrent MDD (ICD-10 F33) may preferentially capture more chronic or severe disease, limiting generalizability to single-episode depression, and we did not stratify by depression severity, chronicity, or treatment adherence. The exclusion of concurrent psychiatric or neurocognitive disorders strengthens internal validity but limits generalizability, as psychiatric comorbidity frequently co-occurs in spine populations. Procedural heterogeneity, including grouping of instrumented and non-instrumented fusions, varying surgical approaches, and inability to distinguish minimally invasive from open techniques within TriNetX, may introduce outcome variability. Patient-reported functional measures (eg, ODI, VAS) and detailed opioid consumption data were not available. Diagnostic surveillance bias is possible, particularly for anxiety and dementia, which may be more readily documented in patients with known depression; newly coded dementia diagnoses within 90 days may reflect recognition of previously undiagnosed conditions rather than true new-onset neurodegeneration. Somatic outcomes such as chest pain, dizziness, and shortness of breath represent symptom-based diagnoses rather than confirmed structural complications. Given the number of outcomes assessed, the possibility of type I error due to multiple comparisons should be considered, as no formal adjustment was applied.

### Conclusions

Preoperative MDD was associated with increased postoperative psychiatric morbidity, somatic symptom diagnoses, emergency department utilization, and inpatient readmissions following elective single-level lumbar fusion, without evidence of increased reoperation or mortality. These findings suggest that addressing spinal pathology alone may be insufficient to optimize postoperative recovery in this population, although this hypothesis requires prospective validation. These results are consistent with existing guideline recommendations to incorporate routine psychiatric risk assessment and perioperative mental health optimization into care pathways for lumbar fusion candidates with MDD.

## Supplemental Material

Supplemental Material - The Impact of Major Depressive Disorder on Somatic and Psychiatric Outcomes Following Elective Single-Level Lumbar Fusion: A Propensity Score-Matched Analysis Supplemental Material for The Impact of Major Depressive Disorder on Somatic and Psychiatric Outcomes Following Elective Single-Level Lumbar Fusion: A Propensity Score-Matched Analysis by Sri Tummala, Hetsinhji Chavda, Tarun R. Sontam, David C. Gibbs, Ioannis Avramis, James M. Rizkalla in Global Spine Journal

## Data Availability

The data that support the findings of this study are available from TriNetX. Restrictions apply to the availability of these data, which were used under license for this study. Data are available from https://trinetx.com with the permission from TriNetX.
