# Supplementary material for: The Impact of Major Depressive Disorder on Somatic and Psychiatric Outcomes Following Elective Single-Level Lumbar Fusion: A Propensity Score-Matched Analysis
Source: Global Spine J. 2026 Apr 24:21925682261445029. Online ahead of print. doi: 10.1177/21925682261445029 (PMC13109243; doi:10.1177/21925682261445029)
Supplement: Supplemental Material - The Impact of Major Depressive Disorder on Somatic and Psychiatric Outcomes Following Elective Single-Level Lumbar Fusion: A Propensity Score-Matched Analysis [file sj-pdf-1-gsj-10.1177_21925682261445029.pdf]

Appendix A: Codes for Inclusion, Exclusion, Matching, and Outcomes

Title	Code	Description
Procedure code- Inclusion	CPT: 22633, 22612, 22558, or 22630	Arthrodesis, Single interspace, Lumbar
Procedure code- Exclusion	CPT: 22634, 22585, 22614, 22632	Arthrodesis, Additional Interspace, lumbar
Inclusion diagnosis	ICD-10: F33	Major depressive disorder, recurrent
Exclusion diagnosis – psychiatric	ICD-10: F01–F03	Dementias (vascular, other, unspecified)
Exclusion diagnosis – psychiatric	ICD-10: F05	Delirium due to known physiological condition
Exclusion diagnosis – psychiatric	ICD-10: F07	Behavioral disorders due to known physiological condition
Exclusion diagnosis – psychiatric	ICD-10: F10–F14	Substance use disorders (alcohol, opioids, cannabis, etc.)
Exclusion diagnosis – psychiatric	ICD-10: F20	Schizophrenia
Exclusion diagnosis – psychiatric	ICD-10: F30–F31	Manic episode and bipolar disorder
Exclusion diagnosis – psychiatric	ICD-10: F40–F41.1	Phobias, panic disorder, generalized anxiety disorder
Exclusion diagnosis – psychiatric	ICD-10: F43.0–F43.2	Acute stress reaction, PTSD, adjustment disorders
Exclusion diagnosis – psychiatric	ICD-10: F44–F45	Dissociative and somatoform disorders
Exclusion diagnosis – psychiatric	ICD-10: F50–F52	Eating, sleep, and sexual dysfunctions
Exclusion diagnosis –	ICD-10: F60	Specific personality disorders

psychiatric		
Propensity score matching – demographics	Age at index	Patient age at time of surgery
Propensity score matching – demographics	F	Women
Propensity score matching – demographics	M	Men
Propensity score matching – demographics	2,054–5	Black or African American
Propensity score matching – demographics	2,106–3	White
Propensity score matching – demographics	1,002–5	American Indian or Alaska Native
Propensity score matching – demographics	2,076–8	Native Hawaiian or other Pacific Islander
Propensity score matching – demographics	2,135–2	Hispanic or Latino
Propensity score matching – demographics	2,186–5	Not Hispanic or Latino
Propensity score matching – demographics	2,131–1	Other race
Propensity score matching – demographics	2,028–9	Asian
Propensity score matching – demographics	UNK	Unknown race
Propensity score matching – diagnosis	ICD-10: I10	Essential (primary) hypertension
Propensity score matching – diagnosis	ICD-10: E78.5	Hyperlipidemia, unspecified
Propensity score matching – diagnosis	ICD-10: E11	Type 2 diabetes mellitus
Propensity score	ICD-10: E10	Type 1 diabetes mellitus

matching – diagnosis		
Propensity score matching – diagnosis	ICD-10: I25	Chronic ischemic heart disease
Propensity score matching – diagnosis	ICD-10: N18	Chronic kidney disease (CKD)
Propensity score matching – diagnosis	ICD-10: E66	Overweight and obesity
Propensity score matching – diagnosis	ICD-10: E03	Other hypothyroidism
Propensity score matching – diagnosis	ICD-10: E28.2	Polycystic ovarian syndrome
Propensity score matching – diagnosis	ICD-10: F17	Nicotine dependence
Propensity score matching – diagnosis	ICD-10: I63	Cerebral infarction
Propensity score matching – diagnosis	ICD-10: Z68	BMI
Propensity score matching – diagnosis	ICD-10: K70–K77	Diseases of liver
Propensity score matching – medication	RxCode: N05A	Antipsychotics
Propensity score matching – medication	RxCode: N06A	Antidepressants
Propensity score matching – medication	RxCode: N02A	Opioids
Propensity score matching – medication	RxCode: N02BF	Gabapentinoids
Propensity score matching – medication	RxCode: MS200	Skeletal muscle relaxants
Propensity score matching – medication	RxCode: CN101	Opioid analgesics
Propensity score	RxCode: CN104	Nonsteroidal anti-inflammatory

matching – medication		drugs
Propensity score matching – laboratory	Lab: 9,083	BMI measurement
Propensity score matching – laboratory	Lab: 9,037	Hemoglobin A1c measurement
Outcome definition	Deceased	Patient status marked as deceased
Outcome definition	CPT: 99281–99285	Emergency department services
Outcome definition	CPT: 1013699	Inpatient or Observation Consultations
Outcome definition	ICD-10: R07.9	Chest pain, unspecified
Outcome definition	ICD-10: R42	Dizziness and giddiness
Outcome definition	ICD-10: R06.02	Shortness of breath
Outcome definition	ICD-10: F41.1	Generalized anxiety disorder
Outcome definition	ICD-10: F43.2	Adjustment disorders
Outcome definition	ICD-10: F20	Schizophrenia
Outcome definition	ICD-10: F10	Alcohol-related disorders
Outcome definition	ICD-10: F43.1	Post-traumatic stress disorder
Outcome definition	ICD-10: F11	Opioid-related disorders
Outcome definition	ICD-10: F01–F04	Vascular, other, unspecified, or amnestic dementia

Reoperation:

Outcome definition	CPT: 22830	Exploration of spinal fusion
Outcome definition	CPT: 22849, 22852	Revision or removal of spinal fixation devices
Outcome definition	CPT: 22612, 22630, 22633	Revision or extension lumbar fusion procedures

Outcome definition	CPT: 63030, 63035	Revision lumbar decompression/discectomy
Outcome definition	ICD-10-PCS: 0SG00*, 0SP00*	Revision of lumbar fusion or removal of spinal instrumentation
